# Supplementary material for: Susceptibility to Colorectal Cancer Based on HSD17B4 rs721673 and rs721675 Polymorphisms and Alcohol Intake among Taiwan Biobank Participants: A Retrospective Case Control Study Using the Nationwide Claims Data
Source: J Pers Med. 2023 Mar 24;13(4):576. doi: 10.3390/jpm13040576 (PMC10146027; doi:10.3390/jpm13040576)
Supplement: Supplementary file 1 [file jpm-13-00576-s001.zip › jpm-2260308-supplementary.pdf]

Table S1. ICD-9-CM codes of the comorbidities which were used to calculate Charlson comorbid-ity index (CCI) values.

The Comorbidities	ICD-9-CM codes
Myocardial infarct	410 and 412
Congestive heart failure	428
Peripheral vascular disease	441, 4439, 7854, V434, and 3848
Cerebrovascular disease	430, 431, 432, 433, 434, 435, 436, 437, and 438
Dementia	290
Chronic pulmonary disease	490, 491, 492, 493, 494, 495, 496, 500, 501, 502, 503, 504, 505, and 5064
Connective tissue disease	7100, 7101, 7104, 7140, 7141, 7142, 71481, and 725
Peptic ulcer disease	531, 532, 533, and 534
Mild liver disease	5712, 5714, 5715, and 5716
Diabetes	2500, 2501, 2502, 2503, and 2507
Diabetes with end organ damage	2504, 2505, and 2506
Hemiplegia or paraplegia	342 and 3441
Moderate or severe renal disease	582, 5830, 5831, 5832, 5833, 5834, 5835, 5836, 5837, 585, 586, and 588
Moderate or severe liver disease	4560, 4561, 4562, 5722, 5723, 5724, 5725, 5726, 5727, and 5728
Metastatic solid tumor	196, 197, 198, and 199
Acquired immunodeficiency syndrome	042, 043, and 044

Table S2. STROBE Statement—checklist of items that should be included in reports of case–control studies.

	Item No	Recommendation	Page No
**Title and abstract**	1	(*a*) Indicate the study's design with a commonly used term in the title or the abstract	1-2
		(*b*) Provide in the abstract an informative and balanced summary of what was done and what was found	2
**Introduction**			
Background/rationale	2	Explain the scientific background and rationale for the investigation being reported	3
Objectives	3	State specific objectives, including any prespecified hypotheses	4
**Methods**			
Study design	4	Present key elements of study design early in the paper	5
Setting	5	Describe the setting, locations, and relevant dates, including periods of recruitment, exposure, follow-up, and data collection	5
Participants	6	(*a*) Give the eligibility criteria, and the sources and methods of case ascertainment and control selection. Give the rationale for the choice of cases and controls	5
		(*b*) For matched studies, give matching criteria and the number of controls per case	5
Variables	7	Clearly define all outcomes, exposures, predictors, potential confounders, and effect modifiers. Give diagnostic criteria, if applicable	5-6
Data sources/ measurement	8*	For each variable of interest, give sources of data and details of methods of assessment (measurement). Describe comparability of assessment methods if there is more than one group	6
Bias	9	Describe any efforts to address potential sources of bias	12
Study size	10	Explain how the study size was arrived at	5, 8
Quantitative variables	11	Explain how quantitative variables were handled in the analyses. If applicable, describe which groupings were chosen and why	6-7
Statistical methods	12	(*a*) Describe all statistical methods, including those used to control for confounding	6-7
		(*b*) Describe any methods used to examine subgroups and interactions	7
		(*c*) Explain how missing data were addressed	NA
		(*d*) If applicable, explain how matching of cases and controls was addressed	5
		(*e*) Describe any sensitivity analyses	NA
**Results**			
Participants	13*	(a) Report numbers of individuals at each stage of study—eg numbers potentially eligible, examined for eligibility, confirmed eligible, included in the study, completing follow-up, and analysed	6
		(b) Give reasons for non-participation at each stage	6
		(c) Consider use of a flow diagram	NA
Descriptive data	14*	(a) Give characteristics of study participants (eg demographic, clinical, social) and information on exposures and potential confounders	8, 20
		(b) Indicate number of participants with missing data for each variable of interest	NA
Outcome data	15*	Report numbers in each exposure category, or summary measures of exposure	8, 20

Main results	16	(*a*) Give unadjusted estimates and, if applicable, confounder-adjusted estimates and their precision (eg, 95% confidence interval). Make clear which confounders were adjusted for and why they were included	8,20-21
		(*b*) Report category boundaries when continuous variables were categorized	8,20-21
		(*c*) If relevant, consider translating estimates of relative risk into absolute risk for a meaningful time period	8,20-21
Other analyses	17	Report other analyses done—eg analyses of subgroups and interactions, and sensitivity analyses	8, 21
**Discussion**			
Key results	18	Summarise key results with reference to study objectives	10
Limitations	19	Discuss limitations of the study, taking into account sources of potential bias or imprecision. Discuss both direction and magnitude of any potential bias	12
Interpretation	20	Give a cautious overall interpretation of results considering objectives, limitations, multiplicity of analyses, results from similar studies, and other relevant evidence	10-11
Generalisability	21	Discuss the generalisability (external validity) of the study results	10-11
**Other information**			
Funding	22	Give the source of funding and the role of the funders for the present study and, if applicable, for the original study on which the present article is based	13

*Give information separately for cases and controls.

Note: An Explanation and Elaboration article discusses each checklist item and gives methodological background and published examples of transparent reporting. The STROBE checklist is best used in conjunction with this article (freely available on the Web sites of PLoS Medicine at http://www.plosmedicine.org/, Annals of Internal Medicine at http://www.annals.org/, and Epidemiology at http://www.epidem.com/). Information on the STROBE Initiative is available at http://www.strobe-statement.org.
